# Intramembrane protease RasP boosts protein production in *Bacillus*

**DOI:** 10.1186/s12934-017-0673-1

**Published:** 2017-04-04

**Authors:** Jolanda Neef, Cristina Bongiorni, Vivianne J. Goosens, Brian Schmidt, Jan Maarten van Dijl

**Affiliations:** 1 Department of Medical Microbiology, University of Groningen, University Medical Center Groningen, Hanzeplein 1, P.O. Box 30001, 9700 RB Groningen, The Netherlands; 2 DuPont Industrial Biosciences, 925 Page Mill Road, Palo Alto, CA 94304 USA; 3 Department of Bioengineering, Centre for Synthetic Biology, Imperial College London, London, SW7 2AZ UK

**Keywords:** *Bacillus*, RasP, Site-2 protease, Secretion, Amylase, Protease

## Abstract

**Background:**

The microbial cell factory *Bacillus subtilis* is a popular industrial platform for high-level production of secreted technical enzymes. Nonetheless, the effective secretion of particular heterologous enzymes remains challenging. Over the past decades various studies have tackled this problem, and major improvements were achieved by optimizing signal peptides or removing proteases involved in product degradation. On the other hand, serious bottlenecks in the protein export process per se remained enigmatic, especially for protein secretion at commercially significant levels by cells grown to high density. The aim of our present study was to assess the relevance of the intramembrane protease RasP for high-level protein production in *B. subtilis*.

**Results:**

Deletion of the *rasP* gene resulted in reduced precursor processing and extracellular levels of the overproduced α-amylases AmyE from *B. subtilis* and AmyL from *Bacillus licheniformis*. Further, secretion of the overproduced serine protease BPN’ from *Bacillus amyloliquefaciens* was severely impaired in the absence of RasP. Importantly, overexpression of *rasP* resulted in threefold increased production of a serine protease from *Bacillus clausii*, and 2.5- to 10-fold increased production of an AmyAc α-amylase from *Paenibacillus curdlanolyticus*, depending on the culture conditions. Of note, growth defects due to overproduction of the two latter enzymes were suppressed by *rasP*-overexpression.

**Conclusion:**

Here we show that an intramembrane protease, RasP, sets a limit to high-level production of two secreted heterologous enzymes that are difficult to produce in the *B. subtilis* cell factory. This finding was unexpected and suggests that proteolytic membrane sanitation is key to effective enzyme production in *Bacillus*.

## Background

Secretory protein production is of critical importance in biotechnology, because this generally delivers high amounts of correctly folded proteins for which downstream processing from the fermentation broth is relatively easy. The Gram-positive bacterium *Bacillus subtilis* and related bacilli are amongst the best protein producers known today and, therefore, frequently used to produce commercially relevant enzymes. In particular, these organisms are suitable for large-scale high-density fermentation, leading to product yields in the 25 g/l range [1]. Thus, they have a long history in the production of, for example, amylases [2, 3] and proteases [2, 4] used in the food, textile and pharmaceutical industries [5]. Another advantage of *B. subtilis* and its close relatives is that they lack toxic by-products, such as endotoxin, which makes them suitable for the Qualified Presumption of Safety (QPS) status of the European Food Safety Authority. Accordingly, many *Bacillus* products have obtained the Generally Recognized As Safe (GRAS) status from the US Food and Drug Administration [6, 7].

Much effort has been made to optimize protein secretion in *B. subtilis*. Major improvements were achieved by optimizing signal peptides [8, 9] or removing proteases involved in product degradation [10]. For example, the deletion of multiple genes for extracellular proteolytic enzymes allowed efficient production not only of technical enzymes, such as a thermostable β-1,3-1,4-glucanase from *Clostridium thermocellum* [11], but also of pharmaceutical proteins, such as single-chain antibodies [12] or human interleukin-3 [13]. Further improvements in protein secretion were achieved at lab-scale by overexpression of the signal peptidase SipS [14], the peptidyl-prolyl *cis*/*trans* isomerase PrsA [15], or the staphylococcal thiol-disulphide oxidoreductase DsbA [16]. Nonetheless, serious bottlenecks in the protein export process per se have remained enigmatic, especially for protein secretion at commercially significant levels by cells grown to high density [17].

Notably, it was previously shown that deleting the gene for the intramembrane protease RasP of *B. subtilis* led to elevated levels of the membrane proteins FtsL [18], HtrA and HtrB, but compromised the production of various other membrane proteins [19] and processing of the α-amylase AmyQ of *Bacillus amyloliquefaciens* [20, 21]. This focused our attention on a possible role of RasP in secretory protein production, especially because the biogenesis of many membrane proteins relies on the general secretory pathway [22]. RasP belongs to the family of site-2 proteases (S2P), more specifically the zink-metallo proteases, which cleave their substrates within the plane of the cytoplasmic membrane [23]. These proteases are conserved in all domains of life where they have roles in regulated intramembrane proteolysis. For instance, the *Escherichia coli* S2P named RseP was shown to cleave signal peptides upon their signal peptidase-mediated liberation from secretory precursor proteins [24, 25]. *B. subtilis* RasP was shown to cleave the anti-sigma factor RsiW under conditions of oxidative- or temperature stress, causing induction of the so-called σ^W^ regulon, which is believed to support cell envelope integrity and to mitigate effects of extracellular stress [20, 21, 26, 27]. Therefore, the present study was aimed at determining whether RasP could be a bottleneck for protein production in *B. subtilis.* Indeed, our results show that RasP overexpression can boost protein production in this important cell factory.

## Methods

### Bacterial strains and growth conditions

The bacterial strains used in this study are listed in Table 1. *B. subtilis* strains were grown at 37 °C, 280 rpm in Lysogeny Broth (LB; Oxoid Limited), MBU medium or 5SM12 medium. The MBU medium is similar to the MBD medium as described by Vogtentanz et al. [28], but lacks soytone and instead of 7.5% glucose it contains 2.1% glucose and 3.5% maltodextrin DE13-17. The 5SM12 medium consists of 75 mM K_2_HPO_4_, 25 mM NaH_2_PO_4_, 12% maltodextrin, 5% Difco Bacto Soytone, 2 mM sodium citrate, 0.5 mM MgSO_4_, 0.2 mM MnCl_2_, 0.03 mM calcium chloride, and 0.0053% ferric ammonium citrate. Growth media were supplemented with neomycin 15 µg/ml or phleomycin 4 µg/ml to select for particular gene deletions. Chloramphenicol was added to 5 or 25 µg/ml for, respectively, the selection of chromosomally integrated amylase or protease expression cassettes and their amplification. In pulse-chase labeling experiments with cells grown on MBU medium, 2.5 µg/ml chloramphenicol was used.

**Table 1:** Bacterial strains used in this study

	Relevant genotypes and phenotypes	Source or reference
*B. subtilis* strain		
*Δupp*	*degUHy32*, *amyE*::*xylRPxylAcomK*-*ermC*, Δ*upp::neoR,* Neo^R^	This study
AmyL	*degUHy32*, *amyE*::*xylRPxylAcomK*-*ermC*, *aprE*::*PaprE*-*amyL catR* T*bpnʹ*; Em^R^; Cm^R^	This study
AmyE	*degUHy32*, *amyE*::*xylRPxylAcomK*-*ermC*, *aprE*::*PaprE*-*amyE catR* T*bpnʹ*; Em^R^; Cm^R^	This study
BPNʹ	*degUHy32*, *amyE*::*xylRPxylAcomK*-*ermC*, *aprE*::*PaprE*-*bpnʹ catR* T*bpnʹ*; Em^R^; Cm^R^	This study
*ΔrasP*	degUHy32, *amyE*::*xylRPxylAcomK*-*ermC*, Δ*upp*::*neoR*, *ΔrasP::upp*-*phleoR*-*cI* Em^R^; Phleo^R^; Neo^S^	This study
AmyL *ΔrasP*	*degUHy32*, *amyE*::*xylRPxylAcomK*-*ermC*, *aprE*::*PaprE*-*amyL catR,* Δ*upp::neoR, ΔrasP::upp*-*phleoR*-*cI catR*; Em^R^; Phleo^R^; Neo^S^ Cm^R^	This study
AmyE *ΔrasP*	*degUHy32*, ∆*scoC*, *amyE*::*xylRPxylAcomK*-*ermC*, ∆*opp, aprE*::*PaprE*-*amyE catR, Δupp::neoR*, *ΔrasP::upp*-*phleoR*-*cI catR*; Em^R^; Phleo^R^; Neo^S^ Cm^R^	This study
BPNʹ *ΔrasP*	*degUHy32*, ∆*scoC*, *amyE*::*xylRPxylAcomK*-*ermC*, ∆*opp, aprE*::*PaprE*-*bpn’ catR*; *Δupp::neoR, ΔrasP::upp*-*phleoR*-*cI* Em^R^; Phleo^R^; Neo^S^ Cm^R^	This study
*ΔtepA*	degUHy32, amyE::*xylRPxylAcomK*-*ermC*, Δ*upp*::*neoR*, *ΔtepA::upp*-*phleoR*-*cI* Em^R^; Phleo^R^; Neo^S^	This study
AmyL *ΔtepA*	*degUHy32*, a*myE*::*xylRPxylAcomK*-*ermC*, *aprE*::*PaprE*-*amyL catR,* Δ*upp::neoR, ΔtepA::upp*-*phleoR*-*cI catR*; Em^R^; Phleo^R^; Neo^S^ Cm^R^	This study
AmyE *ΔtepA*	*degUHy32*, *amyE*::*xylRPxylAcomK*-*ermC*, *aprE*::*PaprE*-*amyE catR, Δupp::neoR*, *ΔtepA::upp*-*phleoR*-*cI catR*; Em^R^; Phleo^R^; Neo^S^ Cm^R^	This study
BPNʹ *ΔtepA*	*degUHy32*, *amyE*::*xylRPxylAcomK*-*ermC*, *aprE*::*PaprE*-*bpnʹ catR*; *Δupp::neoR, ΔtepA::upp*-*phleoR*-*cI* Em^R^; Phleo^R^; Neo^S^ Cm^R^	This study
Properase	*degUHy32*, *amyE*::*xylRPxylAcomK*-*ermC*, *aprE*::*PaprE*-*properase catR* T*bpnʹ*; Em^R^; Cm^R^	This study
Properase RasP	*degUHy32*, *amyE*::*xylRPxylAcomK*-*ermC*, *aprE*::*PaprE*-*properase catR* T*bpnʹ*, *spoIIIAH*::P*spoVG*-*rasP*; Em^R^; Cm^R^	This study
AmyAc	*degUHy32*, *amyE*::*xylRPxylAcomK*-*ermC*, *aprE*::*PaprE*-*amyAc catR* T*bpnʹ*; Em^R^; Cm^R^	This study
AmyAc RasP	*degUHy32*, *amyE*::*xylRPxylAcomK*-*ermC*, *aprE*::*PaprE*-*amyAc catR* T*bpnʹ*, *spoIIIAH*::P*spoVG*-*rasP*; Em^R^; Cm^R^	This study
Plasmids		
pHT315-P_spac_	Multicopy shuttle vector replicating in *E. coli* and *B. subtilis* contains IPTG-inducible P_spac_; Amp^R^, Em^R^	Genencor/dupont [44]
pHTK315	pHT315-P_spac_ derivative, Em^R^ is replaced by Km^R^; Amp^R^, Km^R^	This study
pHT315K::*rasP*	pHT315K-P_spac_ derivative, contains IPTG-inducible *rasP*; Amp^R^, Km^R^	This study

*Tbpnʹ* terminator structure of *B. amyloliquefaciens bpn*ʹ; *Em* ^*R*^ erythromycin resistant; *Phleo* ^*R*^ phleomycin resistant; *Neo* ^*R*^ neomycin resistant; *Neo* ^*S*^ neomycin sensitive; *Cm* ^*R*^ chloramphenicol resistant; *Amp* ^*R*^ ampicillin resistant; *Km* ^*R*^ kanamycin resistant

### Construction of strains and plasmids

Ex Taq polymerase, dNTPs and buffers used for the construction of mutant strains were purchased from Takara Bio Inc. Phusion High Fidelity DNA polymerase (New England Biolabs) was used for the construction of plasmids. Primers were obtained from Eurogentec. Construction of deletion mutants in a *B. subtilis* Δ*upp*::*neo* ^*R*^ strain was performed using the modified mutation delivery method described by Fabret et al. [29]. To delete a particular gene of interest (i.e. *rasP* or *tepA*), its 5′ and 3′ flanking regions were amplified using primer pairs designated P1/P2 and P3/P4 (Table 2). The amplified fragments were fused to a cassette containing a phleomycin resistance marker, the *upp* gene and the *cI* gene [29]. The resulting fusion product was used to transform *B. subtilis* Δ*upp*::*neo* ^*R*^, where competence was induced with 0.3% xylose due to the presence of a xylose-inducible *comK* gene. This resulted in phleomycin resistant and neomycin sensitive strains lacking the target gene. PCRs using primer combinations P0/P4 and P0/CI2.rev (Table 2) were performed to verify the correct deletion. To achieve overproduction and secretion of AmyE [30], AmyL [31] or BPNʹ-Y217L (in short BPNʹ) [32, 33], the promoter of *aprE* (600 bp upstream of the GTG start codon) [34] and the signal sequence of *aprE* were fused to the 7-codon pro-sequence of *amyE*, the *amyL* gene lacking its signal sequence, or the eighth codon of the BPNʹ signal sequence, respectively. For AmyE, the C-terminal starch-binding module was removed by the introduction of a stop codon after the codon for residue 425 in *amyE* and the complete gene expression cassette was produced synthetically (GeneOracle, Santa Clara, CA). Transcription of *amyE* or *bpn*ʹ was terminated using the native BPNʹ terminator, and the native terminator in case of *amyL.* To accomplish the expression and secretion of Properase^®^ (i.e. the subtilisin variant of *Bacillus clausii*) or AmyAc [i.e. an engineered α-amylase from *Paenibacillus curdlanolyticus* that belongs to the AmyAc family (NCBI reference sequence: WP_040711139)], the respective genes were ordered synthetically (GeneArt, Thermofisher Scientific) and fused to the promoter and signal sequence of *aprE* as described above. For transcription termination, the native BPNʹ terminator was used. Genes encoding the five afore-mentioned secretory proteins were integrated into the *aprE* locus by single cross-over recombination using a vector based on plasmid pJH101 [35]. Lastly, gene amplification was achieved by growing transformants at increasing chloramphenicol concentrations up to 25 µg/ml.

**Table 2:** Primers used in this study

Primer name	5′ → 3′ nucleotide sequence
CI2.rev	CTTCAACGCTAACTTTGAG
*rasP*.P0	GCTCTTCAAGGCGAACAGG
*rasP*.P1	CGCCTCATCATTACGGCATC
*rasP*.P2	CGACCTGCAGGCATGCAAGCTACCACCTTATGTGAGTATTGAATTGAC
*rasP*.P3	CGAGCTCGAATTCACTGGCCGTCGGGATACGTCAATTCAATACTCACATAAGGTGGTACGAAAAGTAAATCAATCAGAGGTGC
*rasP*.P4	GATCGTACGGCGCAACG
*tepA*.P0	CGCACGGGCACGATG
*tepA*.P1	CTGTCCGTTCCAGTGTACGG
*tepA*.P2	CGACCTGCAGGCATGCAAGCTCTCGCTTTCATCCTTTCCG
*tepA*.P3	CGAGCTCGAATTCACTGGCCGTCGCAAAGAGAAACTCGGAAAGGATGAAAGCGAGTTCTTTATACCGTGATGCCTCAG
*tepA*.P4	GGTCTGTCATTCAATTTAGACTCCAG
pHT315CPEC.fw	CTATGAGTCGCTTTTTTAAATTTGGAAAGTTAC
pHT315CPEC.rev	CACTGTTTTTAGTCTGTTTCAAAACAGTAG
*kana*CPEC.fw	CTACTGTTTTGAAACAGACTAAAAACAGTGGCTCCGTCGATACTATGTTATACG
*kana*CPEC.rev	TAACTTTCCAAATTTAAAAAAGCGACTCATAGGCTTTTTAGACATCTAAATCTAGGTAC
pHT315*rasP*.fw	GTGAGTATTGAATTGACGTATCCCCGCCCGGGTACCGAGCTCTTAATTG
pHT315*rasP*.rev	GCGAAGAAATGAGACAAAGCTTGACGGCTTGGCGTAATCATGGTCATAGC TG
*rasP*.fw	CGGGGATACGTCAATTCAATACTCAC
*rasP*.rev	CGTCAAGCTTTGTCTCATTTCTTCGC

To complement the Δ*rasP* mutation, plasmid pHTK315, a derivative of pHT315-P_spac_ [36] containing the *kan* gene for kanamycin resistance was constructed using the CPEC strategy described by Quan et al. [37]. Plasmid pHT315-P_spac_ was amplified by PCR using the primers pHT315CPEC.fw and pHT315CPEC.rev. In parallel, the *kan* gene was amplified from the vector pGDL48 [38], using the primers kanaCPEC.fw and kanaCPEC.rev both containing approximately 30 bp overlap with the pHT315-P_spac_ vector. The two resulting PCR fragments were fused and amplified by PCR, and the resulting amplicon was used to transform competent cells of *E. coli* strain TG1. The plasmid thus obtained was named pHTK315. Next, plasmid pHTK315-*rasP* was constructed following the same strategy. For amplification of pHTK315, the primer combination pHT315rasP.fw/pHT315rasP.rev was used, and *rasP* was amplified using the primer combinations rasP.fw/rasP.rev. Both fragments were fused by PCR based on the 30 bp overlaps. The resulting amplicon was used to transform competent cells of *E. coli* TG1, and the plasmid thus obtained was named pHTK315-*rasP*. The correctness of pHTK315 and pHTK315-*rasP* was verified by sequencing and, subsequently, these plasmids were introduced into *B. subtilis* using xylose-induced competence, as described above. After the transformation, mutant strains containing amplified *amyE*, *amyL* or *bpnʹ* expression cassettes were selected by repeated transfers to fresh LB plates containing 25 μg/ml chloramphenicol.

### Analysis of secreted protein production by LDS-PAGE

Cultures were inoculated from LB plates with 25 μg/ml chloramphenicol and grown for approximately 8 h in LB broth with 25 µg/ml chloramphenicol. These cultures were diluted 1000-fold in MBU medium with 2.5 µg/ml chloramphenicol in Ultra Yield Flasks™ (Thomson Instrument Company) and incubated for approximately 16 h at 37 °C, 280 rpm in a Multitron orbital shaker (Infors) at high humidity. After measuring and correcting for the optical density at 600 nm (OD_600_), equal amounts of cells were separated from the culture medium by centrifugation. For the analysis of extracellular proteins, proteins in the culture medium were precipitated with trichloroacetic acid (TCA; 10% w/v final concentration), dissolved in LDS buffer (Life Technologies) and heated for 10 min at 95 °C. Next, proteins were separated by LDS-PAGE on 10% NuPage gels (Life Technologies). Gels were stained with SimplyBlue™ SafeStain (Life Technologies). Gel images were quantified with the ImageJ software (http://imagej.nih.gov/ij/).

### Pulse-chase protein labeling experiments

Pulse-chase labeling of *B. subtilis* proteins was performed using Easy tag [^35^S]-methionine (PerkinElmer Inc.). Immunoprecipitation and LDS-PAGE were performed as described previously [39] using the following adaptations. Cells were grown for 16 h in MBU with 2.5 µg/ml chloramphenicol as described before and diluted 1 h prior to the actual labeling to OD_600_ ~0.7 in fresh MBU with 2.5 µg/ml chloramphenicol. Labeling was performed with 25 µCi [^35^S]-methionine for 30 s before adding an excess amount of unlabeled methionine (chase; 0.6 mg/ml final concentration). Samples were collected at several time points, followed by direct precipitation of the proteins with 10% TCA (w/v) on ice. Precipitates were re-suspended in lysis buffer (10 mM Tris pH 8, 25 mM MgCl_2_, 200 mM NaCl and 5 mg/ml lysozyme). After 10–15 min incubation at 37 °C, lysis was achieved by adding 1% (w/v) SDS and heating for 10 min at 100 °C. Specific polyclonal antibodies against AmyE or AmyL were used for immunoprecipitation of the respective labeled proteins in STD-Tris buffer (10 mM Tris pH 8.2, 0.9% (w/v) NaCl, 1.0% (v/v) triton X-100, 0.5% (w/v) sodium deoxycholate) with the help of Protein A affinity medium (Mabselect Sule, GE Healthcare Life Sciences).

Because of the high proteolytic activity of BPNʹ, which also degrades antibodies, the immunoprecipitation of BPNʹ was performed in the presence of a specific serine protease inhibitor (4 mM, Pefablock SC, Roche). Due to aspecific binding of the BPNʹ antibodies to unidentified cellular proteins of *B. subtilis*, the immunoprecipitation of BPNʹ was only performed to assay secreted BPNʹ in TCA-precipitated culture medium samples. Labeled proteins were separated by LDS-PAGE using 10% NuPage gels (Life Technologies) and visualized using a Cyclon Plus Phosphor Imager (Perkin Elmer). Quantification of the obtained data was achieved by making use of the ImageJ software.

For pulse-chase labeling studies on the complementation of the Δ*rasP* mutation, cells containing pHTK315-*rasP* or control cells containing the empty vector pHTK315 were pre-cultured for 20 h in MBU with 2.5 µg/ml chloramphenicol, because these cells grow slightly slower than cells without such plasmids. One hour prior to labeling with [^35^S]-methionine, the cells were diluted to an OD_600_ of ~0.7 in fresh MBU with 2.5 µg/ml chloramphenicol containing 25 µM isopropyl β-d-1-thiogalactopyranoside (IPTG) necessary for the induction of the P_spac_ promoter. The pulse-chase protein labeling was performed as described above.

### Assays for Properase or AmyAc production

To analyze Properase secretion by cells overexpressing *rasP* or wt control cells, the respective strains were pre-cultured for 5 h in LB at 37 °C. From these pre-cultures 1.5 OD units were used to inoculate 25 ml of MBU medium in Ultra Yield Flasks™, and culturing was continued at 37 °C (250 rpm, 70% humidity). Samples were withdrawn from the cultures at 18, 25, 41, 48 and 65 h of growth for OD_600_ readings and protease activity measurements. OD_600_ was determined using a SpectraMax spectrophotometer (Molecular Devices, Downington, PA, USA). Protease activity in the samples was determined by incubating sample aliquots with the synthetic substrate *N*-Succinyl-Ala-Ala-Pro-Phe *p*-nitroanilide (Sigma Chemical Co) and absorbance readings at 405 nm using a SpectraMax spectrophotometer, as described previously in WO 2010/144283.

To test the effect of *rasP* overexpression on the production of AmyAc, four colonies from the *rasP* overexpressing strain or the wt control strain were used to inoculate LB with 25 µg/ml chloramphenicol and the resulting pre-cultures were grown for 4 h at 37 °C. Next, 0.075 OD units from a pre-culture were used to inoculate 2 ml of medium (5SM12 or MBU) in 24 deep well microtiter plates and cultures were grown for 48 h at 37 °C under vigorous shaking. Samples were withdrawn at 18, 25, 41 and 48 h of culturing for OD_600_ readings and amylase activity measurements. Amylase activity in whole-broth samples was assayed with the Ceralpha HR kit (Megazyme, Wicklow, Ireland) and absorbance readings at 400 nm according to the manufacturer’s instructions.

## Results and discussion

**Fig. 1:**
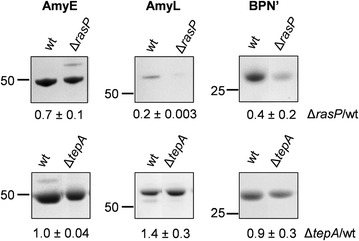
Reduced secretion levels of AmyE, AmyL and BPN’ in *rasP* mutant cells. Δ*rasP* mutant cells and wild-type (wt) or Δ*tepA* mutant control cells expressing AmyE, AmyL or BPNʹ were grown for 16 h in MBU medium. Next, cells and growth media were separated by centrifugation. Proteins in growth medium fractions were precipitated with TCA and analyzed by LDS-PAGE. Protein bands were visualized with the SimplyBlue SafeStain. Molecular weights of marker proteins are indicated (in kDa) on the *left side* of each gel segment. Secreted amounts of AmyE, AmyL and BPNʹ in the growth medium fractions from three independent cultures were assessed by ImageJ analysis of the gels, and the ratios of each of these proteins in the medium fractions of the Δ*rasP* or Δ*tepA* strains relative to the wt strain are indicated below each gel segment together with the standard deviation

**Fig. 2:**
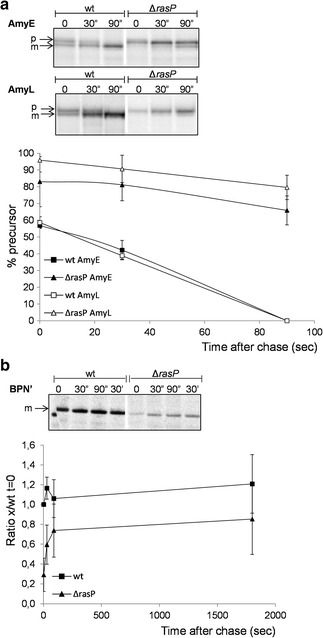
Reduced rates of AmyE, AmyL and BPNʹ secretion in *rasP* mutant cells. **a** Processing of the precursor proteins of AmyE or AmyL by signal peptidase was analyzed by pulse-chase protein labeling with [^35^S]-methionine, immunoprecipitation of AmyE or AmyL from culture samples with specific antibodies, LDS-PAGE and phosphorimaging as described in “Methods”. The positions of precursor (p) and mature (m) forms of AmyE and AmyL are indicated. Data from three independent experiments were analyzed with ImageJ to assess the kinetics of precursor processing, and the results are plotted below the autoradiographs. The *plot* shows the relative amounts (%) of the precursor forms of AmyE (*black symbols*) or AmyL (*white symbols*) in the Δ*rasP* (*triangle*) or wt (*square*) strains at different time points after the chase with non-radioactive methionine (t = 0). *Error bars* show the standard deviation. **b** Secretion of BPNʹ was analyzed by pulse-chase labeling with [^35^S]-methionine, immunoprecipitation from growth medium fractions devoid of cells with specific antibodies, LDS-PAGE and phosphorimaging as described in “Methods”. The position of mature BPNʹ is indicated (m). Data from three independent experiments were analyzed with ImageJ to determine the kinetics BPN’ appearance in the growth medium, and the results are plotted below the autoradiographs. The *plot* shows the average of the calculated ratio of secreted BPNʹ in the Δ*rasP* (*triangle*) or wt (*square*) strains relative to the amount of BPNʹ secreted immediately after the chase with non-radioactive methionine (t = 0) in the wt strain. *Error bars* indicate the standard deviation

**Fig. 3:**
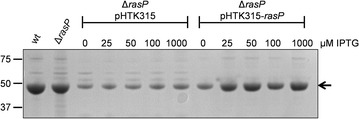
IPTG-dependent complementation of the Δ*rasP* mutation in AmyE-producing cells containing pHTK315-*rasP.* Δ*rasP* mutant bacteria overproducing AmyE and carrying either pHTK315-*rasP* or the empty vector pHTK315 were grown for 16 h in MBU with 2.5 µg/ml chloramphenicol. Next *rasP* expression in cells containing pHTK315-*rasP*, where *rasP* is transcribed from the IPTG-dependent P_spac_ promoter, was induced for 4 h by the addition of IPTG to different end concentrations as indicated. Δ*rasP* mutant bacteria carrying the empty vector pHTK315 were also treated with IPTG as a negative control. Proteins in the growth medium were precipitated with TCA and separated by LDS-PAGE. Protein bands were visualized with the SimplyBlue SafeStain. The AmyE band is indicated with an *arrow*, and the molecular weights of marker proteins are indicated on the *left* of the gel (in kDa)

**Fig. 4:**
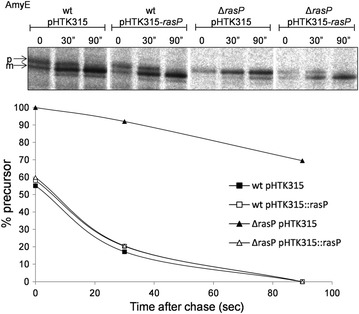
Complementation of pre-AmyE processing in Δ*rasP* mutant cells. Processing of pre-AmyE (p) to mature AmyE (m) was analyzed by pulse-chase protein labeling with [^35^S]-methionine in IPTG-induced Δ*rasP* mutant or wt cells containing either pHTK315-*rasP* or the empty vector pHTK315. Pre-AmyE and mature AmyE were immunoprecipitated with specific antibodies, separated by LDS-PAGE, and visualized by a phosporimaging. Data from two independent experiments were analyzed with ImageJ to assess the kinetics of pre-AmyE processing to the mature form, and the results are plotted below the autoradiographs. Specifically, the *plot* shows the relative amounts (%) of the precursor form of AmyE in Δ*rasP* mutant (*triangle*) or wt (*square*) cells carrying pHTK315-rasP (*white symbols*) or pHTK315 (*black symbols*) at different time points after the chase with non-radioactive methionine (t = 0)

As a first approach to assess the possible function of RasP in protein secretion under fermentation-mimicking conditions, the *rasP* gene was deleted from the *B. subtilis* genome and the secretion of three representative model proteins was assessed in the resulting Δ*rasP* strain. Specifically, the secreted model proteins were the α-amylase AmyE from *B. subtilis*, the α-amylase AmyL from *Bacillus licheniformis* and the serine protease BPNʹ from *B. amyloliquefaciens.* The respective genes were expressed to high levels using the *aprE* promoter, which is a preferred promoter for enzyme production at industrial scale [40]. As shown in Fig. 1, Δ*rasP* cells grown to stationary phase in about 16–20 h of culturing secreted less AmyE, AmyL or and BPNʹ than wild-type cells. This was clearly not the case for control cells lacking the *tepA* gene, which encodes an unrelated cytoplasmic protease [41]. Furthermore, the rates of AmyE and AmyL precursor processing as determined by pulse-chase labeling with [^35^S]-methionine after 16 h of growth were substantially reduced in Δ*rasP* cells compared to wild-type cells (Fig. 2a). In case of BPNʹ we were unable to detect cell-associated precursor forms of this protein but, nonetheless, we showed that the rate of appearance of the mature [^35^S]-methionine-labeled BPNʹ in the growth medium was strongly slowed down in cells lacking *rasP* (Fig. 2b). Together, these findings clearly demonstrate that RasP is needed for efficient processing and secretion of mature AmyE, AmyL and BPNʹ. As illustrated with AmyE, the wild-type rate of precursor processing and secretion of the mature protein were restored when *rasP* was expressed from a plasmid in the Δ*rasP* cells (Figs. 3, 4). This shows that the Δ*rasP* mutation can be complemented with *rasP* expressed in *trans*. Of note, the wild-type cells grew to higher optical densities at 600 nm (OD_600_ ~ 25) than the Δ*rasP* cells (OD_600_ ~ 15), but this effect was corrected for in the loading of gels shown in Figs. 1 and 3, and in the pulse-chase labelling experiments in Figs. 2 and 4 comparable amounts of cells were used.

**Fig. 5:**
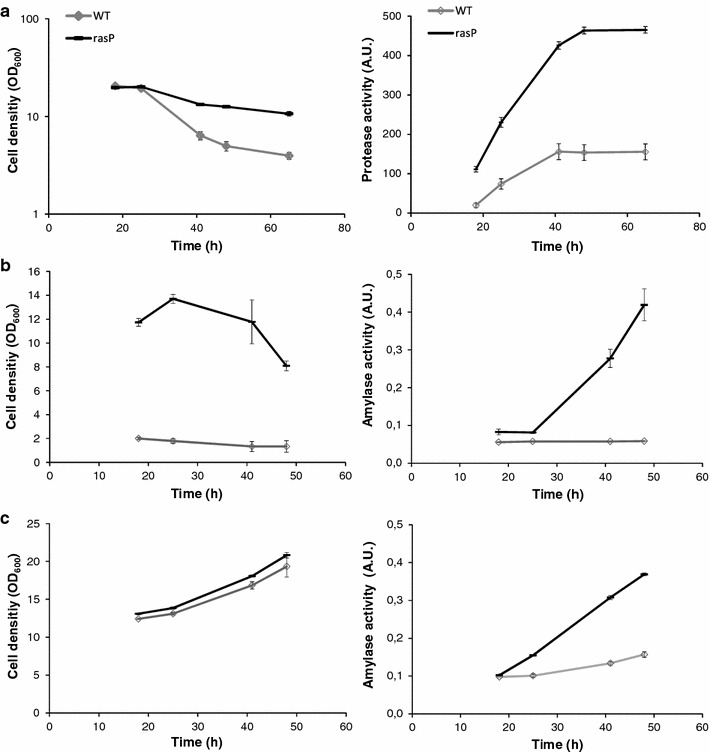
Improved production of Properase and AmyAc upon overexpression of *rasP*. **a** Growth (*left panel*) and extracellular Properase activity (*right panel*) of cells cultured in MBU medium. Measurements on cells that overexpress *rasP* from the strong P*spoVG* promoter are indicated with *black lines* and measurements on wt cells are indicated with *grey lines*. **b** Growth (*left panel*) and extracellular AmyAc activity (*right panel*) of cells that overexpress *rasP* from the P*spoVG* promoter (*black lines*) or wt cells (*grey lines*) cultured in MBU medium. **c** Growth (*left panel*) and extracellular AmyAc activity (*right panel*) of cells that overexpress *rasP* from the P*spoVG* promoter (*black lines*) or wt cells (*grey lines*) cultured in 5SM12 medium. All *plots* in **a**–**c** show average values from three independent experiments, and the *error bars* represent the standard deviations of the respective measurements

Because the removal of RasP had significant influence on the processing and secretion of AmyL, AmyE and BPNʹ, we wanted to know whether *rasP* overexpression could be beneficial for protein secretion in *B. subtilis*. Notably, in ‘wild-type’ *B. subtilis* the secretion of AmyL, AmyE and BPNʹ is already very efficient. Moreover, these enzymes are produced really well in other industrial *Bacillus* species, which gives the optimization of their production in *B. subtilis* a lower commercial impact. Therefore, we focused our attention on two other enzymes, namely a protease (‘Properase’) from *B. clausii* and AmyAc from *P. curdlanolyticus*, which are both commercially valuable but hard to produce. Especially the large-scale production of enzymes of the AmyAc family is very challenging in *Bacillus* species. To overexpress *rasP*, this gene was placed under control of the very strong *spoVG* promoter (P*spoVG*). Importantly, the P*spoVG* promoter is a constitutive promoter and its strength is comparable to that of the promoter of *aprE*, which was used to express the secreted proteins employed in this study (data not shown). Next, the capability of the resulting strain to secrete the highly active Properase, which is even capable of degrading prions, was tested. A potential problem caused by high-level Properase production is a negative effect on the viability of *B. subtilis*. As shown in Fig. 5a, P*spoVG*-driven expression of *rasP* enhanced both cell viability and the secretion of Properase in the production phase when cells were grown in MBU medium. Of note, high-level *rasP* expression led to an increase in Properase production of about threefold. To further obtain proof-of-principle that P*spoVG*-driven *rasP* expression may be beneficial for secretory protein production, we investigated the effect on production of a bacterial α-amylase belonging to the AmyAc family. Similar to Properase, the expression of the AmyAc enzyme had a negative impact on growth in MBU medium and, in this case, both growth and amylase production remained relatively low unless *rasP* was overexpressed (Fig. 5b). In fact, AmyAc production by cells grown in MBU was up to tenfold increased upon *rasP* overexpression. Interestingly, expression of the AmyAc enzyme does not impact on growth in 5SM12 medium, which allowed us to distinguish between growth effects and effects of *rasP* overexpression on production of the secreted AmyAc enzyme. As shown in Fig. 5c, the yield of this enzyme in the 5SM12 growth medium was about 2.5-fold increased, which implies that the improved productivity was mostly directly related to *rasP* overexpression rather than an enhanced cell density of the culture. The main difference between the 5SM12 and MBU media is that the 5SM12 medium contains soytone and 3.4-fold more maltodextrin. This suggests that AmyAc production may have a negative impact on nutrient acquisition by cells grown in MBU, which can be bypassed either by *rasP* overexpression or the provision of soytone and/or additional maltodextrin. Altogether, our results imply that *rasP* overexpression gives significant benefits for producing secretory proteins commercially.

## Conclusions

In conclusion, our present study shows that the S2P intramembrane protease RasP sets the limit to efficient extracellular production of two proteins in *B. subtilis*, namely Properase and an AmyAc type amylase. These proteins are difficult to produce, which is partly due to effects on cell growth and/or viability in late stages of the fermentation process. Enhanced expression of *rasP* can overcome these negative effects, and seems even capable of boosting the secretion of the AmyAc type amylase up to tenfold. Our present findings are unprecedented, giving the first proof-of-principle that overexpression of a protease that cleaves within the plane of the cytoplasmic membrane of a bacterium can lead to improved protein production. The precise mechanism by which RasP exerts this effect in *Bacillus* is not yet known but, based on knowledge from other studies on S2P proteases, we envisage at least three possible scenarios. Firstly, RasP may facilitate the removal of cleaved signal peptides from the membrane [25], secondly, RasP may clear the membrane of mislocalized secretory precursor proteins that may interfere with essential membrane processes [20], or thirdly, overproduced RasP may modulate expression of σ^w^-dependent genes that somehow influence productivity [26]. In these three scenarios, *rasP* overexpression could increase the fitness of the producing cells through the prevention of membrane and cell envelope perturbations. This would then lead to enhanced growth and productivity. A fourth possible explanation would be that RasP activity precludes potentially inhibitory effects of accumulating signal peptides on the SecA preprotein translocation motor [42] and/or on signal peptidases that convert translocated precursors of secretory proteins to the mature form [43]. Of course, combinations of these four scenarios are also conceivable. Irrespective of the precise mechanisms, we conclude that RasP can be applied to boost protein secretion in *Bacillus*, and that the overexpression of this and other S2P proteases represents a promising avenue for future cell factory engineering.

## Abbreviations

GRAS: Generally Recognized As Safe
IPTG: isopropyl β-d-1-thiogalactopyranoside
LDS: lithium dodecyl sulphate
OD_600_: optical density at 600 nm
PAGE: polyacrylamide gel electrophoresis
PCR: polymerase chain reaction
QPS: Qualified Presumption of Safety
S2P: site-2 protease
TCA: trichloroacetic acid

## References

[1] Van Dijl JM, Hecker M (2013). *Bacillus subtilis*: from soil bacterium to super-secreting cell factory. Microb Cell Fact.

[2] Sarvas M, Harwood CR, Bron S, Van Dijl JM (2004). Post-translocational folding of secretory proteins in Gram-positive bacteria. Biochim Biophys Acta.

[3] Palva I (1982). Molecular cloning of alpha-amylase gene from *Bacillus amyloliquefaciens* and its expression in *B. subtilis*. Gene.

[4] Krishnappa L, Dreisbach A, Otto A, Goosens VJ, Cranenburgh R, Harwood CR, Becher D, Van Dijl JM (2013). Extracytoplasmic proteases determining the cleavage and release of secreted proteins, lipoproteins, and membrane proteins in *Bacillus subtilis*. J Proteome Res.

[5] Westers L, Westers H, Quax WJ (2004). *Bacillus subtilis* as cell factory for pharmaceutical proteins: a biotechnological approach to optimize the host organism. Biochim Biophys Acta.

[6] Olempska-Beer ZS, Merker RI, Ditto MD, DiNovi MJ (2006). Food-processing enzymes from recombinant microorganisms—a review. Regul Toxicol Pharmacol.

[7] Earl AM, Losick R, Kolter R (2008). Ecology and genomics of *Bacillus subtilis*. Trends Microbiol.

[8] Brockmeier U, Caspers M, Freudl R, Jockwer A, Noll T, Eggert T (2006). Systematic screening of all signal peptides from *Bacillus subtilis*: a powerful strategy in optimizing heterologous protein secretion in Gram-positive bacteria. J Mol Biol.

[9] Caspers M, Brockmeier U, Degering C, Eggert T, Freudl R (2010). Improvement of Sec-dependent secretion of a heterologous model protein in *Bacillus subtilis* by saturation mutagenesis of the N-domain of the AmyE signal peptide. Appl Microbiol Biotechnol.

[10] Pohl S, Bhavsar G, Hulme J, Bloor AE, Misirli G, Leckenby MW, Radford DS, Smith W, Wipat A, Williamson ED, Harwood CR, Cranenburgh RM (2013). Proteomic analysis of *Bacillus subtilis* strains engineered for improved production of heterologous proteins. Proteomics.

[11] Luo Z, Gao Q, Li X, Bao J (2014). Cloning of LicB from *Clostridium thermocellum* and its efficient secretive expression of thermostable beta-1,3-1,4-glucanase. Appl Biochem Biotechnol.

[12] Wu XC, Ng SC, Near RI, Wong SL (1993). Efficient production of a functional single-chain antidigoxin antibody via an engineered *Bacillus subtilis* expression-secretion system. Biotechnology (N Y).

[13] Westers L, Dijkstra DS, Westers H, van Dijl JM, Quax WJ (2006). Secretion of functional human interleukin-3 from *Bacillus subtilis*. J Biotechnol.

[14] Bolhuis A, Sorokin A, Azevedo V, Ehrlich SD, Braun PG, De Jong A, Venema G, Bron S, Van Dijl JM (1996). *Bacillus subtilis* can modulate its capacity and specificity for protein secretion through temporally controlled expression of the *sipS* gene for signal peptidase I. Mol Microbiol.

[15] Kontinen VP, Sarvas M (1993). The PrsA lipoprotein is essential for protein secretion in *Bacillus subtilis* and sets a limit for high-level secretion. Mol Microbiol.

[16] Kouwen TR, van der Goot A, Dorenbos R, Winter T, Antelmann H, Plaisier MC, Quax WJ, van Dijl JM, Dubois JY (2007). Thiol-disulphide oxidoreductase modules in the low-GC Gram-positive bacteria. Mol Microbiol.

[17] Chen J, Fu G, Gai Y, Zheng P, Zhang D, Wen J (2015). Combinatorial Sec pathway analysis for improved heterologous protein secretion in *Bacillus subtilis*: identification of bottlenecks by systematic gene overexpression. Microb Cell Fact.

[18] Bramkamp M, Weston L, Daniel RA, Errington J (2006). Regulated intramembrane proteolysis of FtsL protein and the control of cell division in *Bacillus subtilis*. Mol Microbiol.

[19] Zweers JC, Wiegert T, van Dijl JM (2009). Stress-responsive systems set specific limits to the overproduction of membrane proteins in *Bacillus subtilis*. Appl Environ Microbiol.

[20] Heinrich J, Hein K, Wiegert T (2009). Two proteolytic modules are involved in regulated intramembrane proteolysis of *Bacillus subtilis* RsiW. Mol Microbiol.

[21] Heinrich J, Lundén T, Kontinen VP, Wiegert T (2008). The *Bacillus subtilis* ABC transporter EcsAB influences intramembrane proteolysis through RasP. Microbiology.

[22] Yuan J, Zweers JC, van Dijl JM, Dalbey RE (2010). Protein transport across and into cell membranes in bacteria and archaea. Cell Mol Life Sci.

[23] Dalbey RE, Wang P, van Dijl JM (2012). Membrane proteases in the bacterial protein secretion and quality control pathway. Microbiol Mol Biol Rev.

[24] Akiyama Y, Kanehara K, Ito K (2004). RseP (YaeL), an *Escherichia coli* RIP protease, cleaves transmembrane sequences. EMBO J.

[25] Saito A, Hizukuri Y, Matsuo E, Chiba S, Mori H, Nishimura O, Ito K, Akiyama Y (2011). Post-liberation cleavage of signal peptides is catalyzed by the site-2 protease (S2P) in bacteria. Proc Natl Acad Sci USA.

[26] Zweers JC, Nicolas P, Wiegert T, Van Dijl JM, Denham EL (2012). Definition of the σ (W) regulon of *Bacillus subtilis* in the absence of stress. PLoS ONE.

[27] Helmann JD (2016). *Bacillus subtilis* extracytoplasmic function (ECF) sigma factors and defense of the cell envelope. Curr Opin Microbiol.

[28] Vogtentanz G, Collier KD, Bodo M, Chang JH, Day AG, Estell DA, Falcon BC, Ganshaw G, Jarnagin AS, Kellis JT, Kolkman MA, Lai CS, Meneses R, Miller JV, de Nobel H, Power S, Weyler W, Wong DL, Schmidt BF (2007). A *Bacillus subtilis* fusion protein system to produce soybean Bowman–Birk protease inhibitor. Protein Expr Purif.

[29] Fabret C, Ehrlich SD, Noirot P (2002). A new mutation delivery system for genome-scale approaches in *Bacillus subtilis*. Mol Microbiol.

[30] Yang M, Galizzi A, Henner D (1983). Nucleotide sequence of the amylase gene from *Bacillus subtilis*. Nucleic Acids Res.

[31] Yuuki T, Nomura T, Tezuka H, Tsuboi A, Yamagata H, Tsukagoshi N, Udaka S (1985). Complete nucleotide sequence of a gene coding for heat- and pH-stable alpha-amylase of *Bacillus licheniformis*: comparison of the amino acid sequences of three bacterial liquefying alpha-amylases deduced from the DNA sequences. J Biochem.

[32] Wells JA, Ferrari E, Henner DJ, Estell DA, Chen EY (1983). Cloning, sequencing, and secretion of *Bacillus amyloliquefaciens* subtilisin in *Bacillus subtilis*. Nucleic Acids Res.

[33] Wells JA, Cunningham BC, Graycar TP, Estell DA (1987). Recruitment of substrate-specificity properties from one enzyme into a related one by protein engineering. Proc Natl Acad Sci USA.

[34] Valle F, Ferrari E, Smith I, Slepecky AR, Selow P (1989). Subtilisin: a redundantly temporally regulated gene. Regulation of prokaryotic development.

[35] Perego M, Sonenshein AL, Hoch JA, Losick R (1993). Integrational vectors for genetic manipulation in *Bacillus subtilis*. *Bacillus subtilis* and other Gram-positive bacteria: biochemistry, physiology, and molecular genetics.

[36] Fukushima T, Furihata I, Emmins R, Daniel RA, Hoch JA, Szurmant H (2011). A role for the essential YycG sensor histidine kinase in sensing cell division. Mol Microbiol.

[37] Quan J, Tian J (2009). Circular polymerase extension cloning of complex gene libraries and pathways. PLoS ONE.

[38] Meijer WJ, de Jong A, Bea G, Wisman A, Tjalsma H, Venema G, Bron S, van Dijl JM (1995). The endogenous *Bacillus subtilis* (natto) plasmids pTA1015 and pTA1040 contain signal peptidase-encoding genes: identification of a new structural module on cryptic plasmids. Mol Microbiol.

[39] Van Dijl JM, De Jong A, Smith H, Bron S, Venema G (1991). Non-functional expression of *Escherichia coli* signal peptidase I in *Bacillus subtilis*. J Gen Microbiol.

[40] Ferrari E, Valle F. Mutant aprE promoter. Genencor. 2000; EP1244794 B1.

[41] Bolhuis A, Matzen A, Hyyryläinen HL, Kontinen VP, Meima R, Chapuis J, Venema G, Van Dijl JM (1999). Singal peptide peptidase- and ClpP-like proteins of *Bacillus subtilis* required for efficient translocation and processing of secretory proteins. J Biol Chem.

[42] Cunningham K, Wickner W (1989). Specific recognition of the leader region of precursor proteins is required for the activation of translocation ATPase of *Escherichia coli*. Proc Natl Acad Sci USA.

[43] Wickner W, Moore K, Dibb N, Geissert D, Rice M (1987). Inhibition of purified *Escherichia coli* leader peptidase by the leader (signal) peptide of bacteriophage M13 procoat. J Bacteriol.

[44] Worner K, Szurmant H, Chiang C, Hoch JA (2006). Phosphorylation and functional analysis of the sporulation initiation factor Spo0A from *Clostridium botulinum*. Mol Microbiol.

